# Colloidal/Calcified *Neurocysticercosis* at University Hospital of KSA: A Case Series

**DOI:** 10.4314/ejhs.v33i3.20

**Published:** 2023-05

**Authors:** Saima Nazish, Mohammad Almuhanna

**Affiliations:** 1 Department of Neurology, College of medicine, Imam Abdulrahman Bin Faisal University, Dammam, Saudi Arabia; 2 College of medicine, King Faisal University, Dammam, Saudi Arabia

**Keywords:** Neurocysticercosis, Seizures, Neuroimaging, Kingdom of Saudi Arabia

## Abstract

**Background:**

Neurocysticercosis (NCC) is considered the most common central nervous system (CNS) helminthic infection. The prevalence of NCC cases in the Middle East has increased in recent years. Thirty-nine cases of NCC were reported between 2003-2011 in the Arabian Peninsula, among, five cases being from the kingdom Saudi Arabia (KSA). Most of the cases reported from the KSA were presented with seizures, and they belonged to expatriate workers or their related contacts. In this case series, we presented three cases of colloidal/calcified NCC.

**Cases:**

Our patients were diagnosed with NCC based on epidemiologic exposure, clinical features, and typical radiological findings. Stool samples for ova and parasites were negative in all three cases. Among these cases, two patients were immigrants and belonged to endemic areas, and third case who is the youngest in this series was a Saudi, without any history of exposure to any source.

**Results:**

The first and the third cases were treated with Albendazole and Dexamethasone. We did not offer any medication regarding NCC in the second case as he had asymptomatic NCC and the disease was inactive so did not mandate anti-helminth medication.

**Conclusion:**

NCC in KSA, like in non-endemic countries, is not a rare or unusual infection anymore, presenting with seizures or incidental findings in an asymptomatic state. Vigilant diagnostic protocols with efficient diagnostic tools are required for detecting carriers of the adult form of the parasite. Timely detection of these carriers can avoid further spread and its related complications in the Saudi population.

## Introduction

The most common helminthic infection of the central nervous system (CNS) is *Neurocysticercosis* (NCC). Taenia solium tapeworm is acquired by ingestion of pork contaminated by larval cysts. The tapeworms live for a few years, during which the tapeworm carrier sheds ova. The ovas are spread from person to person. After ingestion of the ova, the larva hatch and spread through the bloodstream to the tissues (1). It can affect any tissue of the body, eyes, muscles, and subcutaneous tissues other than CNS causing NCC. The major manifestations of NCC include seizures, headaches, and elevated intracranial pressure. Blindness, meningitis, and dementia are unusual (2). Endemic areas of NCC include East, South, and Southeast Asia, Sub-Saharan Africa, and Latin America. Globalization and increasing rates of immigration have contributed to the increased incidence of NCC in high-income countries (3). Similarly, the prevalence of NCC cases in the Middle East has increased in recent years (4). Thirty-nine cases of NCC were reported between 2003-2011 in Arabian Peninsula, among these, five cases were from KSA (5). Most of the cases reported from KSA belonged expatriate workers and their related contacts, presented with seizures (6,7). Here, we are presenting three cases of NCC in a young age group of different nationalities presented to our hospital with seizures and managed accordingly.

**Figure 1:**
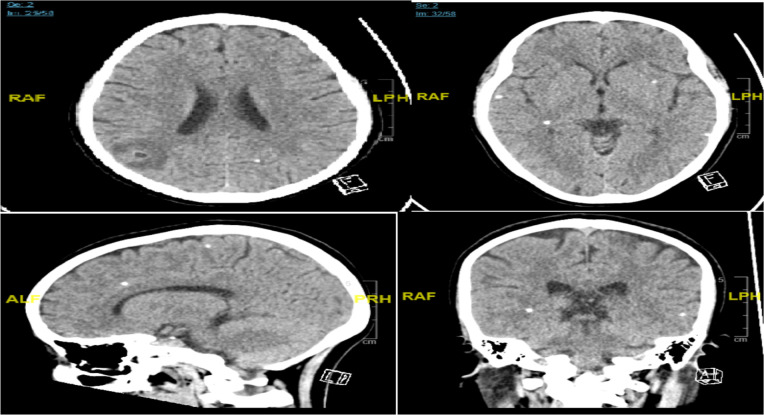
Multiple hyperdense small area in bilateral hemisphere mainly in the gray white matter area. Right parieto-occipital hypodense area with grade I perilesional vasogenic edema with no mass effect.

**CASE 1:** Our first case was a 36 years old Vietnamese female who presented to the emergency department with a history of headache for 1 day followed by three attacks of generalized tonic-clonic seizures, completely recovered to normal consciousness. There was no focality on neurological examination, and systemic examination was unremarkable. Computed tomography (CT) brain (Figure 1) showed findings of NCC confirmed by magnetic resonance imaging (MRI) study, which showed multiple small lesions located at the right parietal area which demonstrates low signal intensity in T2 weighted sequence with peripheral smooth conglomerated enhancement and mild perilesional edema. EEG recording did not show any epileptiform or non-epileptiform abnormality. Routine blood investigations including, inflammatory markers and CSF analysis were normal. Stool analysis did not reveal any ova and parasites. Bilateral thigh (femur) X-rays showed elongated calcifications within the soft tissue “Rice Grain” appearance mainly right thigh. For seizures, Tab levitiracetam 250 mg bid was started. For her active NCC lesion, Cap albendazole 300mg PO Q12H for 14 days and Dexamethasone 4mg per oral daily were started after consultation with on call infectious disease consultant. She did not develop any further episodes of seizures and was discharged from the hospital for OPD follow-up.

**Figure 2:**
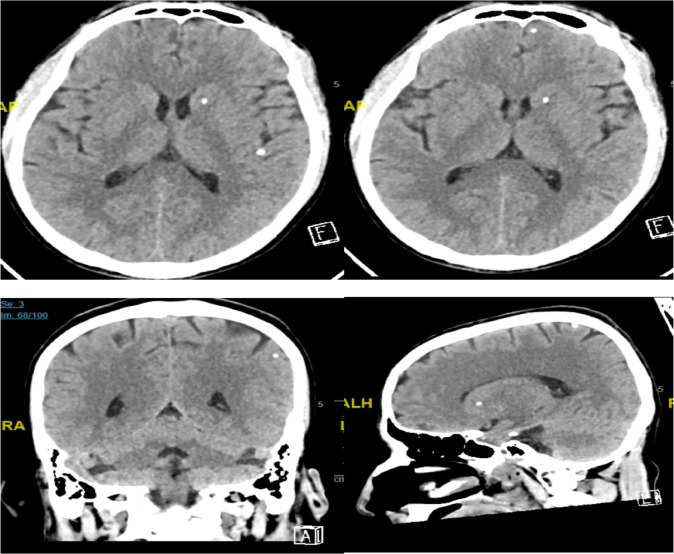
Multiple supratentorial intra-and extra axial foci of calcification likely related to nodular calcified neurocysticercosis.

**CASE 2:** Our second case was a 27 years old male from Nepal, brought to the emergency department by the Red Crescent as a victim of a deep neck laceration injury, as a result of a suicidal attempt in an unstable condition. After initial stabilization, neck exploration, and repair, Psychiatrist evaluated the patient on suspicion of Major depression disorder with catatonia and psychosis A routine Head CT showed NCC findings; detailed neurological examination was unremarkable. EEG recording did not show any epileptiform or non-epileptiform abnormality. Head MRI with contrast (Figure 2) showed confirmed NCC findings of multiple foci of intracranial calcification seen within the left caudate head, left frontal, bilateral temporal, right occipital, and left parietal lobes with an absence of adjacent perifocal edema as well as the absence of pathological enhancement. Stool ova and parasite 3 consecutive samples were negative. The infectious disease team did not offer any medication regarding NCC as it is inactive and asymptomatic and does not mandate anti-helminth medication. The patient was discharged from hospital in a stable condition.

**Figure 3:**
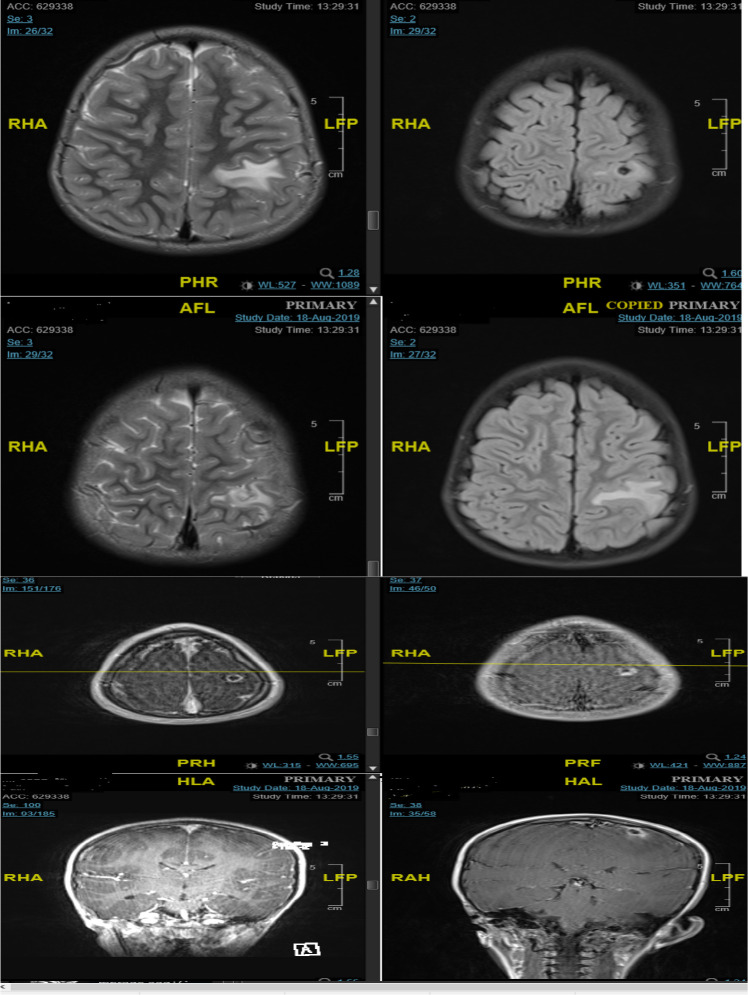
Left postcentral gyrus cortical lesion which measures 0.7 x 0.9 cm and display high T2, low T1 signal with no diffusion restriction. FLAIR images demonstrate low signal intensity with central foci of high signal. Furthermore, Perifocal edema seen involving post central gyrus. No other lesion identified. No evidence of blooming. Post contrast images demonstrate uniform wall ring enhancement.

**CASE 3:** This case belonged to a 6-year-old Saudi girl who was admitted due to right focal onset epilepsy with right focal clonic seizures followed by Todd's paresis and right sensory seizures in our hospital. On clinical examination, patient was vitally stable, Afebrile had Glasgow Coma Scale (GCS) of 15/15 with normal neurological examination. MRI brain (Figure 3) showed a left postcentral gyrus lesion of 0.7 x 0.9 cm with uniform wall ring enhancement and perifocal edema (hyperintense on T2 and hypointense on T1 with no diffusion restriction). This lesion was suggested as NCC. EEG showing multifocal epileptiform discharges. Stool analysis and cultures were negative for parasites. She was managed initially with phenytoin 85 mg PO TID; and antiparasitic medications were started-Albendazole 300 mg PO Q12, Dexamethasone 4mg/PO/Q12hr for 2 weeks.

Within the first day of the albendazole treatment, and despite the steroid; the patient exhibited signs of cortical irritation with uprising abdominal sensation and occasional paresthesia in the limbs. Her anti-epileptic regimen was upgraded. Later on, she was discharged from the hospital in a stable condition. Moreover, follow-up EEG did not reveal any abnormality.

## Discussion

In this case series, we presented three cases Of NCC. The patients were presented to our hospital with clinical manifestations, varying from an asymptomatic state to headache and focal onset with bilateral tonic-clonic seizures. These clinical presentations are similar with what is reported in literature. Seizures and headaches are listed on top of the most frequent NCC's clinical presentation (2). NCC is usually diagnosed on clinical manifestations, epidemiologic exposure and typical neuroimaging findings, like calcified and cystic lesions with an eccentric ‘dot’ representing the scolex. Important radiological differentials of NCC include tuberculomas, hydatid cysts, gliomas, and metastatic lesions. Our patients were diagnosed based on epidemiologic exposure, clinical features, and typical radiological findings. Stool samples for ova and parasites were negative in all three cases, as stool examination is also not very sensitive enough due to the time lag between the appearance of clinical manifestations and taenia solium eggs exposure. Two patients from this series were immigrants and belonged to endemic areas like Nepal and Vietnam. A large case series of NCC was recently reported from Kuwait and the Middle East, where they retrospectively reviewed all confirmed NCC cases.

Among 150 diagnosed cases, 98 were expatriates, belonging to different taeniasis-endemic countries, mostly India (55 of 150, 36.7%) and Nepal (12 of 150, 8%). According to this case series, immigrants from India and nearby countries are most common source of carrying the infection to the Arab world; most local population are acquiring NCC from immigrants who work in different services. They also detect a cluster of families with multiple family members who acquired infection from household workers. Most of the clinical and radiological characteristics of NCC were similar with other parts of the world. Seizures or persistent headache was the most common presentation.

Radiologically, 59% of cases showed lesions in their frontal lobes, followed by 30.7% in the occipital lobes (4). Previously, a case NCC in an Indian female immigrant was reported from our center. The patient was presented with new-onset seizures, in a post-partum state. Later on, she was diagnosed with NCC as a cause of her seizure (7), Suggesting that NCC should also be considered as an important differential diagnosis in patients presenting with seizures, especially if they are from disease-endemic areas. Similarly, a cross-sectional study related to the etiology and diagnosis of patients with epilepsy reported that NCC was the most commonly identified cause of epilepsy in the Asian subgroup of patients (9).

Our third case who is the youngest in this series was a Saudi, without any history of exposure to the source. Previously, NCC in an 18th-month-old child was reported from KSA, who acquired infection from domestic workers (6). Autochthonous transmission was observed in cases reported from the Arabian Peninsula, where the majority having occurred in wealthy families who employed household workers from disease-endemic areas. These persons were carriers and transferred the infection through fecal-oral routes (4,5). In one study, high levels of anti-T. solium taeniasis-specific IgG antibodies were detected in around 4.8% of the blood samples of the newly arrived domestic workers. Among the total 120 stool samples screened, five samples were found positive for infection caused by Taenia species (4.16%) (8) Similarly, in one large series of Middle East patients, Autochthonous transmission was the underlying cause of most cases, through contact to individuals from disease-endemic areas (4). Among the cytocidal therapies, Albendazole (15 mg/kg/day) is used as the first-line antiparasitic medication given for 10-14 days. Dual therapy with albendazole and praziquantel (50 mg/kg/day) with adjunctive corticosteroid therapy is recommended for multiple cysts. Our 3rd case showed exacerbation of neurological exacerbation, which is usually expected during the early phase of treatment. The steroid helps in the avoidance of neurological exacerbation (10).

Anti-parasitic therapy is not recommended in patients with untreated elevated intracranial pressure and hydrocephalus or the presence of calcified lesions only. Hence, our second case patient was not given antiparasitic therapy.

In conclusion, NCC in a non-endemic country like KSA is not a rare or unusual infection anymore; the presentation is varying from asymptomatic to parenchymal disease with neurological manifestations including seizures. Medical education and social awareness about the routes of transmission, good personal hygiene with frequent hand-washing of food handlers are still the prime and most effective ways to reduce the transmission. Vigilant diagnostic protocols with efficient diagnostic tools are required for detecting carriers of the adult form of the parasite. Timely detection of these carriers can avoid further spread and its related complications in the Saudi population.
